# Bone Mineral Density and Body Composition of Adult Premenopausal Women with Three Levels of Physical Activity

**DOI:** 10.1155/2013/953271

**Published:** 2013-02-25

**Authors:** Fernando D. Saraví, Fabiana Sayegh

**Affiliations:** ^1^Bone Densitometry Unit, Nuclear Medicine School, 5500 Mendoza, Argentina; ^2^Institute of Physiology, Faculty of Medical Sciences, Nacional University of Cuyo, 5500 Mendoza, Argentina; ^3^Area of Gynecology and Obstetrics, Faculty of Medical Sciences, Nacional University of Cuyo, 5500 Mendoza, Argentina; ^4^University Hospital, Nacional University of Cuyo, 5500 Mendoza, Argentina

## Abstract

Weight-bearing and resistance physical activities are recommended for osteoporosis prevention, but it is unclear whether an intensity level above current recommendations has a positive effect on adult premenopausal women. Body composition and bone mineral density (BMD) by DXA were compared in three groups of women as follows: Sedentary, Maintenance exercise, and federated Sport Team (*n* = 16 for each group). Physical activity was estimated from the International Physical Activity Questionnaire (IPAQ). The groups did not differ in age, height, weight, or body mass index. Bone mineral content and non-fat soft tissue mass were higher and fat mass was lower in the Sport Team group than in the other groups. The same was true for BMD of total skeleton, lumbar spine, femoral neck, and total hip. A test for linear trend of body composition and BMD showed significant results when including all three groups. Simple and multiple regression analyses showed significant associations between physical activity level (or alternatively, years of participation in programmed physical activity) and bone mass measures at all sites except for the middle third of radius. It is concluded that a level of physical activity higher than that usually recommended benefits bone health in adult premenopausal women.

## 1. Introduction

Osteoporosis is a major public health problem worldwide [1]. Although there are effective treatments, primary prevention, mostly based on lifestyle changes, remains an essential goal to prevent both osteoporosis and its most serious consequence, namely, fragility fractures. Lifestyle changes include keeping a low alcohol intake, abstaining from smoking, maintaining adequate calcium, vitamin D, and protein intake, and, last but not least, increasing physical activity [2].

Regularly performing weight-bearing and resistance exercises is a major lifestyle measure for osteoporosis prevention [3]. Physical activity may increase peak bone mass in children and adolescents [4]. In postmenopausal women, regular physical activity is useful for improving muscle strength and preventing falls, which is important because most fragility fractures are related to falls [2]. The role of exercise in young adult women has been less explored [5], but current evidence suggests that it has a role in maintaining or even augmenting bone mass [6, 7]. 

Although the importance of physical activity is clearly emphasized by most guidelines, some of these fail to address what its desirable frequency and duration are [8, 9]. A brochure from the National Institutes of Health states the following: “According to the Surgeon General, the optimal goal is at least 30 minutes of physical activity on most days, preferably daily” [10]. The International Osteoporosis Foundation gives several examples of exercise schedules, one of which is “45 to 60 minutes of weight-bearing aerobic exercise three days per week (i.e., brisk walking)” [2]. However, it is not yet clear whether higher levels of physical activity may be associated with additional benefits for bone health, particularly in young adult women. On the other hand, a syndrome characterized by disordered eating, amenorrhea, and osteoporosis, known as the “female athlete triad,” has been described in physically active young women [11, 12].

To address this area of uncertainty, the present study compared bone mineral content (BMC), bone mineral density (BMD), and body composition in adult premenopausal women with three levels of physical activity. 

## 2. Materials and Methods

### 2.1. Participants

This cross-sectional, observational study included 48 women divided in three groups, namely, sedentary women (Sedentary, *n* = 16), women who regularly practiced exercise for maintenance or leisure (Maintenance, *n* = 16), and women who belonged to a sport team (Sport Team, *n* = 16). The participants of the latter group were recruited from nonprofessional, federated volleyball teams. The participants of the other two groups were recruited from women attending a gynecological office, direct contact at the Nuclear Medicine School, and word of mouth.

All participating women were healthy nonsmokers, had menstrual cycles within the normal range, and had completed high school (some were university students or graduates). None was taking oral contraceptives or other drugs known to affect BMD. None reported drinking more than 50 g of alcohol per week. 

The study was planned and conducted in compliance with the current (2008) version of the Declaration of Helsinki [13]. The purpose of the study was explained both orally and through a written document to each woman, who then signed an informed consent if she agreed to participate. The study protocol was reviewed and approved by the Committee of Teaching and Research of the Nuclear Medicine School.

### 2.2. Assessment of Body Composition and Bone Mass

Body composition and bone mass were measured by dual-energy X-ray absorptiometry (DXA) with a Lunar Prodigy equipment (GE Lunar Health Systems, Madison, WI) in the Bone Densitometry Unit of the Nuclear Medicine School. Total body, lumbar spine (L1–L4), left hip, and forearm (middle third of radius at the nondominant side = 33% radius) scans were all performed by the same highly trained technician and analyzed by one of the authors (F. D. Saraví). 

Long-term stability of the scanner was evaluated through daily measurement of a spine phantom, as recommended by the manufacturer (CV < 0.5% for BMD). Short-term precision was assessed by DXA scans repeated after repositioning the subject (two measures at each site in 30 patients) as recommended by the International Society for Clinical Densitometry [14]. For the technician who performed the scans for this study, short-term precision was 0.8% for total body, 1.0% for lumbar spine, 1.4% for femoral neck, and 1.1% for total hip.

Body composition results are expressed as total BMC, total body fat, total non-fat soft tissue mass (NFSTM), fat as a percentage of body weight, total body fat mass index, and total NFSTM index. The latter were calculated, respectively, as the quotient of total body fat (kg) and NFSTM (kg), each divided by height (m) squared [15, 16].

### 2.3. Anthropometric Measures and Assessment of Calcium Intake and Menstrual Cycle

Participant height and weight was measured at the time of the DXA scans with a stadiometer and a clinical scale with a precision of 1 mm and 100 g, respectively. The body mass index (BMI) was calculated as weight (kg) divided by height (m) squared. Calcium intake was evaluated through a validated survey [17]. Participants were asked about frequency, duration, and regularity of their menses. 

### 2.4. Assessment of Physical Activity

All participants answered about their physical activity in the previous seven days with the self-administered, long version of the International Physical Activity Questionnaire (IPAQ) [18] available in Spanish [19]. The results were processed and analyzed according to the guidelines of the IPAQ website [20]. Results are reported as metabolic equivalent minutes per week (MET-min/week). A MET of 4 was assigned to maintenance and leisure physical activities, and a MET of 6 was assigned to volleyball playing [21].

### 2.5. Statistical Analysis

Data were analyzed with the commercial statistical software Prism 5.04 for Windows and InStat3 (GraphPad, San Diego, CA). The D'Agostino and Pearson omnibus normality test was routinely used to assess whether data departed significantly from a Gaussian distribution. If this was the case, data are presented as median (25–75 interquartile range). Otherwise, data are expressed as mean ± standard deviation. Differences between groups for normally distributed data were assessed by one-way ANOVA followed by Tukey's test and a test for linear trend. Data departing significantly from a normal distribution were analyzed by Kruskall-Wallis followed by Dunn's multiple comparison test. Simple linear regression was used to assess the relationship between each of the variables physical activity, duration of practice, age, body weight, BMI, total fat mass, total NFSTM, total body fat mass index, and total NFSTM index on one hand and total BMC and BMD at total body, lumbar spine, femoral neck, total hip, and forearm (33% radius) on the other. The variables showing significant correlation with simple linear regression were then included in a multiple regression analysis with physical activity, duration of practice, body weight, BMI, NFSTM, fat mass, NFSTM index, and fat mass index as independent variables, to find out which of them were best correlated with BMC and BMD. A value of *P* < 0.05 was deemed significant.

## 3. Results and Discussion

There was no significant difference in age, height, weight, BMI, or calcium intake among the three groups, while their physical activity level was significantly different (Table 1). The maintenance group had programmed physical activity with a median of 3 h/week (interquartile range 2 to 3 h/week, range 2 to 4 h/week) during 3.4 ± 1.7 years, while the Sport Team group had a median of 6 h/week (interquartile range 5 to 6 h/week, range 4 to 6 h/week) during 6.5 ± 1.8 years. The small range of time devoted to physical activity by the Sports group is due to the fact that they trained together. The small range of time devoted to physical activity by the Maintenance group is due to the study design, since we wished to assess a moderately active group.

 The results of body composition analysis are summarized in Table 2. Although the groups did not differ in BMI, differences were found for all variables, with significant linear trends when all three groups were compared. However, the mean values of BMC, fat mass, NFSTM, % body fat, fat mass index, and NFSTM index were not significantly different between the Sedentary and the Maintenance groups. BMD results, shown in Table 3, showed significant linear trends for the three groups, and the mean values of the Sport Team were significantly higher (except for 33% radius) than those of the Sedentary and Maintenance groups, which in turn did not differ between them.

 For simple linear regression, physical activity and duration of practice were selected as independent variables because they were germane to the study. The influence of age was analyzed because the sample included women with ages ranging from 21 to 46 years. While some studies reported no significant influence of age on BMD within this age range [22, 23], other studies have shown an inverse correlation between age and BMD at the lumbar spine, femoral neck, and forearm [24], or a direct correlation between age and lumbar spine BMD [25, 26] and a negative correlation at the femoral neck [26]. Additionally, the age at which peak BMD is reached may differ between sites and different populations [27–30]. Body weight [31–33] and BMI [31, 34, 35] are strong predictors of BMD. NFSTM is also positively correlated with BMD in young women [32, 36–40]. The influence of fat mass on BMD in this age group is more controversial, since different groups have reported no association [32], a positive association [37–39], or a negative association [41] (see [42] for a recent review). Fat mass index and NFSTM index were analyzed as measures of fat mass and NFSTM, respectively, normalized for height [15].

 Simple linear regression results are shown in Table 4. The age of the participants had no correlation with BMC or BMD, except for a slight negative influence on femoral neck BMD. Fat mass had no correlation with BMC or BMD at any site except for a weak positive relationship with 33% radius BMD; neither the former nor the latter were significant for fat mass index. On the other hand, NFSTM and NFSTM index were significantly correlated with BMC and with BMD at all sites. Body weight was positively and significantly related with BMC and BMD at the lumbar spine and 33% radius, but not femoral neck nor total hip BMD. Physical activity level and time on programmed physical activity (set at 0 for the sedentary group) were significantly correlated with all bone parameters except for 33% radius BMD. 

 The main results of multiple regression analysis are featured in Table 5. The coefficient of determination (*R* ^2^) depicts the fraction of total variance of the dependent variable which is explained by the model.

It can be seen that either the level physical activity or duration of activity (the number of years during which that level had been sustained) is included in all models. It was not possible to include physical activity and duration of activity simultaneously in the models because of colinearity (*R* = 0.823). The highest values of *R* ^2^ were found for the models incorporating physical activity or duration of practice plus body weight and for those incorporating physical activity or duration of practice plus NFSTM plus fat mass. Similar results were obtained when body weight was replaced by BMI, although with generally lower *R* ^2^ values. This is in agreement with a study suggesting that BMI is inferior to body weight as a predictor of BMD [43]. In the models incorporating NFSTM and fat mass, the influence of fat mass was larger for total body BMC, lumbar spine, and 33% radius, while NFSTM was more important than fat mass for total body BMD. 

Limitations of this study include those inherent to cross-sectional comparison, a relatively small sample, and the fact that physical activity was measured indirectly, through the IPAQ instrument.

The Sport Team group differed from the Sedentary and Maintenance groups in all measures of body composition. They had higher BMC and NFSTM and lower fat mass than both the Sedentary and the Maintenance groups. The same was found for BMD of total skeleton, lumbar spine, femoral neck, and total hip. 

However, no significant difference in means other than in physical activity level was found between the Sedentary and the Maintenance groups. This may be due in part to the small sample size, since tests for linear trend including all three groups were significant for all measures except for 33% radius BMD. Additionally, both in simple regression and multiple regression, it was found that both physical activity level and duration of practice had a strong correlation with BMC and total skeleton, lumbar spine, femoral neck, and total hip BMD, but not with 33% radius BMD. 

While it has been suggested that high-intensity resistance training has site-specific effects on BMD; namely, it increases lumbar spine, but not femoral neck BMD [44], a more recent meta-analysis indicates that this may not be the case [7]. In the present series, a robust difference for both femoral neck and total hip BMDs was found between the Sport Team group and both the Sedentary and Maintenance groups. This observation agrees with findings of a recent population-based study of more than one thousand 25-year-old women reporting recreational exercise, in whom a larger BMD difference was found in the femoral neck than in the lumbar spine [45]. Another study found that recreational football increased volumetric BMD in the tibia after a 14-week training course [46].

The importance of combining both regularity and impact for highest positive effects on bone mass has been emphasized [45]. In the present study, both the years during which programmed physical activity was performed and its level of intensity were significantly associated with measures of bone mass, except at 33% radius. Thus, while for older patients the best practical advice may be to walk 30 to 60 min per day on most days [47], younger women can benefit from more demanding schedules combining high impact and regularity—not just in bone health [3, 48]. The characteristics of training for the federated Sport Team participants were such that it should not raise particular concern for the “female athlete triad” [11, 12], although it is advisable to screen all physically active women for this condition [49].

## 4. Conclusion

A higher than usually recommended level of physical activity for adult premenopausal women is associated with higher BMC and NFSTM, lower body fat mass, and higher BMD. Therefore, regarding bone health (and other possible health benefits), young women should be encouraged to engage in that level, provided they are willing to make it regularly.

## Figures and Tables

**Table 1:** Characteristics of participants^a^.

	Sedentary (*n* = 16)	Maintenance (*n* = 16)	Sport Team (*n* = 16)	*P* ^b^
Age (years)	33.5 ± 5.8	34.0 ± 5.0	32.4 ± 8.5	0.7770
Height (cm)	161.0 ± 5.0	161.0 ± 3.0	164 ± 6.0	0.1681
Weight (kg)	61.6 ± 6.5	57.5 ± 5.3	60.5 ± 8.7	0.2434
Body mass index (kg/m^2^)	23.5 ± 1.8	22.2 ± 1.8	22.5 ± 2.5	0.1738
Calcium intake (mg/day)^c^	500 (400–800)	400 (400–800)	800 (400–1100)	0.1026
Physical activity (MET-min/week)	485 ± 152	667 ± 127^d^	1115 ± 175^e^	<0.0001

^a^Values are mean ± standard deviation except for calcium intake, which is median (interquartile range).

^b^Assessed by ANOVA, except for calcium intake, in which Kruskall-Wallis nonparametric test was used.

^c^The ranges of calcium intake were (in mg/day) 200 to 1000 for the Sedentary group, 100 to 1200 for the Maintenance group, and 400 to 1200 for the Sports group.

^d^Different from Sedentary (*P* < 0.01)

^e^Different from Sedentary and Maintenance (both *P* < 0.001).

**Table 2:** Body composition results.

	Sedentary (*n* = 16)	Maintenance (*n* = 16)	Sport Team (*n* = 16)	Linear trend *P* ^a^
Bone mineral content (g)	2295 ± 260	2431 ± 321	2730 ± 375^b^	0.0004
Fat mass (kg)	21.23 ± 3.59	17.71 ± 5.18	15.68 ± 5.42^c^	0.0021
Non-fat soft tissue mass (kg)	38.07 ± 4.37	37.74 ± 3.75	42.55 ± 3.98^d^	0.0030
Body fat (%)	34.3 ± 3.9	30.3 ± 7.3	25.2 ± 5.6^b^	<0.0001
Fat mass index (kg/m^2^)	8.2 ± 1.3	6.8 ± 2.0	5.8 ± 1.8	0.0004
Non-fat soft tissue mass index (kg/m^2^)	14.6 ± 1.2	14.6 ± 1.2	15.9 ± 1.0	0.0045

^a^For linear trend post hoc test after a significant result with ANOVA.

^b^Significantly different from Sedentary (*P* < 0.01) and Maintenance (*P* < 0.05).

^c^Significantly different from Sedentary and Maintenance (both *P* < 0.01).

^d^Significantly different from Sedentary (*P* < 0.01).

^e^Significantly different from Sedentary (*P* < 0.05) and Maintenance (*P* < 0.01).

**Table 3:** Bone mineral density results.

Site	Sedentary (*n* = 16)	Maintenance (*n* = 16)	Sport Team (*n* = 16)	Linear trend *P* ^a^
Total body	1.117 ± 0.069	1.132 ± 0.071	1.220 ± 0.087^b^	0.0004
Lumbar spine	1.153 ± 0.107	1.189 ± 0.125	1.358 ± 0.177^b^	0.0001
Femoral neck	0.942 ± 0.103	0.950 ± 0.102	1.153 ± 0.186^b^	<0.0001
Total hip	0.932 ± 0.085	0.957 ± 0.110	1.159 ± 0.180^b^	<0.0001
33% radius	0.686 ± 0.053	0.681 ± 0.042	0.705 ± 0.072	Not calculated^c^

^a^For linear trend post-hoc test after a significant result with ANOVA.

^b^Significantly different from Sedentary and Maintenance (both *P* < 0.01).

^c^For the 33% radius site, *P* for ANOVA was 0.4606.

**Table 4:** Simple linear regression results for all three groups (*n* = 48).

Independent variable	BMC (g)	BMD total skeleton (g/cm^2^)	BMD lumbar spine (g/cm^2^)	BMD femoral neck (g/cm^2^)	BMD total hip (g/cm^2^)	BMD 33% radius (g/cm^2^)
Age (years)	*R* = 0.0308	*R* = 0.0173	*R* = 0.0958	*R* = 0.3170	*R* = 0.2057	*R* = 0.0936
	*P* = 0.8352	*P* = 0.9074	*P* = 0.5173	*P* = 0.0281	*P* = 0.1601	*P* = 0.5268
Duration of practice (years)	*R* = 0.4523	*R* = 0.4576	*R* = 0.4516	*R* = 0.5202	*R* = 0.5394	*R* = 0.1555
	*P* = 0.0012	*P* = 0.0011	*P* = 0.0013	*P* = 0.0002	*P* < 0.0001	*P* = 0.2913
Physical activity (MET-min/week)	*R* = 0.5065	*R* = 0.4704	*R* = 0.4919	*R* = 0.5303	*R* = 0.5563	*R* = 0.1026
	*P* = 0.0002	*P* = 0.0007	*P* = 0.0004	*P* = 0.0001	*P* < 0.0001	*P* = 0.4876
Weight (kg)	*R* = 0.6055	*R* = 0.3407	*R* = 0.3315	*R* = 0.2012	*R* = 0.1573	*R* = 0.4433
	*P* < 0.0001	*P* = 0.0178	*P* = 0.0214	*P* = 0.1702	*P* = 0.2858	*P* = 0.0016
BMI (kg/m^2^)	*R* = 0.3500	*R* = 0.1876	*R* = 0.2282	*R* = 0.1391	*R* = 0.1031	*R* = 0.3607
	*P* = 0.0147	*P* = 0.2017	*P* = 0.1188	*P* = 0.3456	*P* = 0.4859	*P* = 0.0118
Body fat mass (kg)	*R* = 0.2730	*R* = 0.0151	*R* = 0.0965	*R* = 0.0896	*R* = 0.1127	*R* = 0.3158
	*P* = 0.0606	*P* = 0.9188	*P* = 0.5143	*P* = 0.5449	*P* = 0.4457	*P* = 0.0288
NFSTM (kg)	*R* = 0.5667	*R* = 0.4696	*R* = 0.3548	*R* = 0.3762	*R* = 0.3348	*R* = 0.2864
	*P* < 0.0001	*P* = 0.0008	*P* = 0.0133	*P* = 0.0084	*P* = 0.0200	*P* = 0.0484
Body fat mass index (kg/m^2^)	*R* = 0.1447	*R* = 0.0689	*R* = 0.0250	*R* = 0.1375	*R* = 0.1555	*R* = 0.2475
	*P* = 0.3263	*P* = 0.2194	*P* = 0.8861	*P* = 0.3514	*P* = 0.2912	*P* = 0.0899
NFSTM Index (kg/m^2^)	*R* = 0.3189	*R* = 0.3956	*R* = 0.2593	*R* = 0.3528	*R* = 0.3291	*R* = 0.1803
	*P* = 0.0271	*P* = 0.0054	*P* = 0.0751	*P* = 0.0139	*P* = 0.0224	*P* = 0.2201

BMC: total bone mineral content; BMD: bone mineral density; BMI: body mass index; NFSTM: non-fat soft tissue mass.

*R*: Pearson's coefficient of correlation. Values of *P* indicate the probability of the slope between the independent and the dependent variable being nonsignificantly different from zero.

**Table 5:** Selected multiple regression results for all three groups (*n* = 48).

Independent variables	BMC (g)	BMD total skeleton (g/cm^2^)	BMD lumbar spine (g/cm^2^)	BMD femoral neck (g/cm^2^)	BMD total hip (g/cm^2^)	BMD 33% radius (g/cm^2^)
Physical activity and body weight	*R* ^2^ = 0.568	*R* ^2^ = 0.309	*R* ^2^ = 0.323	*R* ^2^ = 0.334	*R* ^2^ = 0.320	*R* ^2^ = 0.200
	*P* < 0.0001	*P* = 0.0002	*P* = 0.0002	*P* = 0.0001	*P* = 0.0002	*P* = 0.0066
Duration of practice and body weight	*R* ^2^ = 0.629	*R* ^2^ = 0.358	*R* ^2^ = 0.345	*R* ^2^ = 0.358	*R* ^2^ = 0.335	*R* ^2^ = 0.236
	*P* = 0.0004	*P* < 0.0001	*P* < 0.0001	*P* < 0.0001	*P* = 0.0001	*P* = 0.0024
Physical activity and BMI	*R* ^2^ = 0.410	*R* ^2^ = 0.272	*R* ^2^ = 0.314	*R* ^2^ = 0.314	*R* ^2^ = 0.332	*R* ^2^ = 0.147
	*P* < 0.0001	*P* = 0.0008	*P* = 0.0002	*P* = 0.0002	*P* = 0.0001	*P* = 0.0275
Duration of practice and BMI	*R* ^2^ = 0.426	*R* ^2^ = 0.302	*R* ^2^ = 0.323	*R* ^2^ = 0.344	*R* ^2^ = 0.347	*R* ^2^ = 0.192
	*P* < 0.0001	*P* = 0.0002	*P* = 0.0002	*P* < 0.0001	*P* < 0.0001	*P* = 0.0083
Physical activity, NFSTM, and fat mass	*R* ^2^ = 0.555	*R* ^2^ = 0.315	*R* ^2^ = 0.317	*R* ^2^ = 0.303	*R* ^2^ = 0.317	*R* ^2^ = 0.195
	*P* < 0.0001	*P* = 0.0008	*P* = 0.0007	*P* = 0.0011	*P* = 0.0007	*P* = 0.0215
Duration of practice, NFSTM, and fat mass	*R* ^2^ = 0.622	*R* ^2^ = 0.346	*R* ^2^ = 0.346	*R* ^2^ = 0.327	*R* ^2^ = 0.329	*R* ^2^ = 0.249
	*P* < 0.0001	*P* = 0.0002	*P* = 0.0003	*P* = 0.0005	*P* = 0.0005	*P* = 0.0052

BMC: total bone mineral content; BMD: bone mineral density; BMI: body mass index; NFSTM: non-fat soft tissue mass. The units of the independent variables are the same indicated in Table 4.

*R* ^2^: coefficient of determination. Values of *P* correspond to the probability of *R* ^2^ of attaining the displayed value or a higher one by chance.
